# *Pinus massoniana* somatic embryo maturation, mycorrhization of regenerated plantlets and its resistance to *Bursaphelenchus xylophilus*

**DOI:** 10.3389/fpls.2023.1130471

**Published:** 2023-05-09

**Authors:** You-Mei Chen, Qi Fei, Xin-Rui Xia, Xin Ke, Jian-Ren Ye, Li-Hua Zhu

**Affiliations:** ^1^ Jiangsu Key Laboratory of Pest Invasion Prevention and Control, Collaborative Innovation Center of Sustainable Forestry in Southern China, Nanjing, China; ^2^ Institute of Forest Protection, College of Forest, Nanjing Forestry University, Nanjing, China

**Keywords:** *Pinus massoniana*, *Bursaphelenchus xylophilus*, somatic embryogenesis, maturation, germination, ectomycorrhizal fungi

## Abstract

Pine wilt disease, caused by the pine wood nematode (PWN, *Bursaphelenchus xylophilus*), is a major quarantine forest disease that poses a threat to various pine species, including *Pinus massoniana* (masson pine), worldwide. Breeding of PWN-resistant pine trees is an important approach to prevent the disease. To expedite the production of PWN-resistant *P. massoniana* accessions, we investigated the effects of maturation medium treatments on somatic embryo development, germination, survival, and rooting. Furthermore, we evaluated the mycorrhization and nematode resistance of regenerated plantlets. Abscisic acid was identified as the main factor affecting maturation, germination, and rooting of somatic embryos in *P. massoniana*, resulting in a maximum of 34.9 ± 9.4 somatic embryos per ml, 87.3 ± 9.1% germination rate, and 55.2 ± 29.3% rooting rate. Polyethylene glycol was identified as the main factor affecting the survival rate of somatic embryo plantlets, with a survival rate of up to 59.6 ± 6.8%, followed by abscisic acid. Ectomycorrhizal fungi inoculation with *Pisolithus orientalis* enhanced the shoot height of plantlets regenerated from embryogenic cell line (ECL) 20-1-7. Ectomycorrhizal fungi inoculation also improved the survival rate of plantlets during the acclimatization stage, with 85% of mycorrhized plantlets surviving four months after acclimatization in the greenhouse, compared with 37% non-mycorrhized plantlets. Following PWN inoculation, the wilting rate and the number of nematodes recovered from ECL 20-1-7 were lower than those recovered from ECL 20-1-4 and 20-1-16. The wilting ratios of mycorrhizal plantlets from all cell lines were significantly lower than those of non-mycorrhizal regenerated plantlets. This plantlet regeneration system and mycorrhization method could be used in the large-scale production of nematode-resistance plantlets and to study the interaction between nematode, pines, and mycorrhizal fungi.

## Introduction

The pine wilt disease (PWD), caused by the pine wood nematode (PWN, *Bursaphelenchus xylophilus*), is a devastating forest disease, resulting in significant economic and ecological losses (Ye, 2019; Li et al., 2021). Currently, PWD is considered a quarantine disease in 52 countries worldwide (Wu et al., 2020). In China, this disease has rapidly spread to 19 provinces and 742 counties since its first report in Nanjing, Jiangsu Province, by the end of 2021 (Li et al., 2022b). PWN can naturally infest 51 pine species worldwide and at least 17 species in China, including *Pinus massoniana* Lamb. (Ye and Wu, 2022). *P. massoniana* is a conifer species native to southern China, and has both economic and ecological value. It is a major timber species and a pioneer species for afforestation of barren mountains (Fu et al., 1999). Currently, there is no economic viable method to control PWD in infested forests. Resistance breeding is an important way to prevent damage from pests and pathogens to forest trees (Xu et al., 2013; Zhu et al., 2019), and research into this approach is being carried out in China. In 2001, long-term resistance breeding of *P. massoniana* to pine wood nematodiasis was initialed in Anhui Province, China (Cai et al., 2005). A total of 1,201 seedlings from 251 families was selected from 324 healthy mother trees, and a resistance seed orchard was established (Xu et al., 2013). Additionally, artificial inoculation of 40 P*. massonian* accessions in South China was carried out, resulting in the selection of three resistant accessions (GX2, GX3 and GD5) (Xu et al., 2000). However, traditional breeding methods like these are time-consuming, limited by the season, and slow in terms of trait improvement speed.

Plant somatic embryogenesis (SE) is a powerful tool in plant biotechnology, providing a convenient way to achieve rapid propagation of elite genotypes (Pullman et al., 2015). Successful SE has been reported in at least 36 species of *Pinus* spp., with the majority being economically important timber species such as *Pinus densiflora* (Kim and Moon, 2014), *Pinus elliottii* (Yang et al., 2020), *Pinus pinaster* (Arrillaga et al., 2019), *Pinus radiata* (Montalban and Moncalean, 2019), *Pinus taeda* (Pullman et al., 2015) and *Pinus thunbergii* (Sun et al., 2019b). As one of the most important afforestation tree species in China, *P. massoniana* has received extensive attention in SE and plantlet regeneration studies The first SE system for *P. massoniana* was established using mature zygotic embryos as explants, resulting in the regeneration of three plantlets (Huang et al., 1995). Another system using immature zygotic embryos as explants was later established by Yang et al. (2011). In recent years, the embryonal mass initiation, proliferation, maturation and germination of somatic embryos from this species have been further optimized (Chen et al., 2019; Xia et al., 2021). However, despite the progress made in *P. massoniana* SE, several challenges still remain, including low frequency of embryonal mass initiation, low number and poor quality of mature embryos, and low plantlet regeneration rates (Xia et al., 2021). The maturation treatment has been found to affect somatic embryo germination (Salaj et al., 2019), making it important to investigate the effects of different maturation treatments on somatic embryo germination and subsequent plant growth (Jiang et al., 2021). Acclimatization stage is also considered a major obstacles to successful micropropagation of conifers (Mohammed and Vidaver, 1988). Regenerated plantlets grown in sterile tissue culture conditions lack interaction with microorganisms, making successful culture and subsequent acclimatization under non-sterile conditions difficult, leading to poor survival rates after transplantation (Zhu et al., 2010).

Mycorrhiza refers to a beneficial between plants and fungi that colonize their roots (Niemi et al., 2004). In nature, the roots of most terrestrial plants are colonized by mycorrhizal fungi, with pine species predominantly forming ectomycorrhizas (Niemi et al., 2004; Niemi et al., 2007; Zhu et al., 2010). Experimental studies have demonstrated that inoculating of roots with ectomycorrhizal fungi can promote soil water and nutrients absorption (Chen et al., 2016; Garcia et al., 2016), enhance plant tolerance to drought (Gehring et al., 2017), salinity (Guo et al., 2022) and heavy metal stress (Yang et al., 2022) and increase resistance to pests and diseases (Wang et al., 2022). Therefore, early inoculation of ectomycorrhizal fungi onto *in vitro* grown plantlets is an important method to obtain plantlets capable of withstanding various stresses. Previous studies have investigated the potential effects of ectomycorrhizal fungi on certain traits of tree species before and during the transplanting stage of regenerated plantlets (Cheng et al., 1995; Niemi and HäGgman, 2002; Zhu et al., 2010; Wang et al., 2015; Chen et al., 2022). However, the effect of ectomycorrhizal fungi on plantlets regenerated from somatic embryos of *P. massoniana* has not been reported to date.

The plant tissue culture technique is a powerful biotechnological approach that can be used not only for rapid clonal propagation, but also for the study of various tree diseases, and can accelerate pest and disease resistance breeding (Fenning, 2019). This technique provides a relatively simple system that uses organs, tissues, or cells to study the interaction between the pathogen or pest and the host (Jang and Tainter, 1990). Fenning (2019) reviewed 66 publications reporting attempts to generate disease-resistant plantlets using tissue culture methods. These studies involved 30 forest and other tree species and approximately 31 different plant pathogens were identified.

Various studies have utilized plant tissue culture technique to study the interaction between plant pathogens or pests and their hosts. For instance, Jang and Tainter (1990) demonstrated differential resistance to *Phytophthora cinnamomi*, the little leaf disease pathogen, in calli obtained from *Pinus* species. Similarly, Terho et al. (2000) evaluated the growth reactions of 27 Scots pine embryogenic cell lines to *Gremmeniella abietina*, the *Scleroderris* canker fungus. Nagy et al. (2005) found that the embryogenic cell lines of *Picea abies* responded to *Ceratocystis polonica*, the blue-stain fungal pathogen, and *Heterobasidion annosum*, the butt rot pathogen, in a manner similar to that of trees with varying susceptibility to these diseases. Furthermore, Hashmi et al. (1993) investigated the response of peach cultures to *Meloidogyne incognita*, the root-knot nematode, and identified several that exhibited greater resistance than the parental cells. More recently, studies have confirmed the pathogenicity of aseptic PWN by inoculating them onto pine callus or micropropagated plantlets generated *in vitro* (Lin et al., 2017). In addition, Zhu et al. (2019) selected two clones with relatively high resistance levels to PWN by inoculating regenerated *P. densiflora* microshoots with PWN under aseptic conditions. However, there are no reports to date on the PWN resistance of regenerated plantlets of *P. massoniana*.

This study aimed to analyze the primary factors that influence somatic embryo development and maturation in *P. massoniana* as well as investigate the impact of maturation treatments on somatic embryo germination, survival, and root development. Furthermore, the formation of ectomycorrhizae in regenerated plantlets and its influence on PWN resistance in three embryonic cell lines of *P. massoniana* was evaluated *in vitro*.

## Materials and methods

### Initiation, maintenance and proliferation of callus tissue

*P. massoniana* embryogenic callus were initiated from immature zygotic embryos, following the methodology described by Xia et al. (2021). All initiation media contained LP basic medium (Von Arnold and Eriksson, 1977), supplemented with 2,4-dichlorophenoxyacetic acid (2,4-D), 6-benzylaminopurine (6-BA), 0.25 mg/L vitamin C (VC), 30.0 g/L maltose, 1.0 g/L inositol, 0.50 g/L casein hydrolysate, 0.45 g/L glutamine and 6.24 g/L agar, with a pH value adjusted to 5.80 ± 0.02. Six initiation media were generated in the form of different combinations of 2,4-D and 6-BA (**Table 1**). Depending on the availability of seeds, 10-85 explants were cultured per seed family (GX1, GX2, GX3 and GX4). Cultures were grown in the dark at 24 ± 1 °C. Frequency of SE initiation was assessed after two months of culture. The callus tissue was cultured and transferred to proliferation medium, which contained 1.0 mg/L 2,4-D, 0.5 mg/L 6-BA and 15.0 g/L maltose, whereas the concentrations of the other components were the same as in the initiation medium. Embryogenic lines were maintained by every two weeks subcultures.

**Table 1:** Plant growth regulator combinations in initiation medium.

Medium number	2,4-dichlorophenoxyacetic acid (mg/L)	6-benzylaminopurine (mg/L)
1	1.0	0.5
2	1.0	1.0
3	1.5	0.5
4	1.5	1.0
5	2.0	0.5
6	2.0	1.0

### Cytological observation of callus tissue

Callus tissue was observed under a Zeiss stereo microscope, SteRo Discovery v20 (Carl Zeiss, Germany) after staining with 2% (w/v) acetocarmine for 30 s and 0.05% (w/v) Evans blue for 5 s on glass slides (Gupta and Holmstrom, 2005; Montalbán et al., 2012; Xia et al., 2021).

### Maturation of somatic embryos

The effect on somatic embryo maturation of maturation medium containing combinations of abscisic acid (ABA), polyethylene glycol 8000 (PEG 8000), activated carbon (AC) and maltose was tested. The embryogenic tissues were subcultured in darkness for at least one week in liquid medium before being transferred to the maturation medium. This liquid medium was made up of LP medium with reduced concentrations of plant growth regulators (PGRs) (0.5 mg/L 2,4-D and 0.25 mg/L 6-BA), 15.0 g/L maltose, 1.0 g/L inositol, 0.50 g/L casein hydrolysate and 0.45 g/L glutamine. To study maturation, aliquots (3.5 mL) of the cell suspension were dispersed on sterilized filter paper, which was placed on the surface of the solid maturation medium. Each maturation medium treatment was conducted with at least five biological replicates, each represented by one petri dish. All petri dishes were incubated in the dark at 24 ± 1°C. After 75 days of culture, the number of somatic embryos at the cotyledonary stage was recorded.

### Effects of ABA, PEG 8000, AC and maltose concentrations on SE maturation

To test the influence of ABA, PEG 8000, AC, and maltose concentrations on SE maturation efficiency, one embryogenic cell line (ECL) 1-3-5 was evaluated on nine maturation media. The treatments with different concentrations of ABA, PEG 8000, AC and maltose were arranged in a completely randomized design (**Table 2**). Orthogonal arrays with four factors and three concentrations of each were used to identify the primary and secondary factors affecting somatic embryo maturation and the optimal combination. The range analysis method was used to calculate the range of each group of factors to determine the primary and secondary order of the influence of each factor on somatic embryo maturation and identify the optimal strategy.

**Table 2:** Orthogonal array of three maturation media consisting of three concentrations of each of ABA, PEG, AC and maltose.

Levels	Factors			
	ABA (mg/L)	PEG (g/L)	AC (g/L)	Maltose (g/L)
1	1	110	0.5	20
2	2	130	1.0	25
3	3	150	1.5	30

ABA, abscisic acid; PEG, polyethylene glycol 8000; AC, activated carbon.

### Effect of PEG 8000 concentration on SE maturation

To determine the effect of PEG 8000 concentration on mature embryos, aliquots of a cell suspension produced as described earlier were placed on maturation medium containing 2.0 mg/L ABA, 0.5 mg/L gibberellic acid (GA_3_), 0.25 mg/L VC, 25 g/L maltose, 1.0 g/L myo-inositol, 1.0 g/L l-glutamine, 1.0 g/L AC and 3.5 g/L Phytagel, with PEG 8000 at different concentrations, namely 70, 90, 110, 130, 150 or 170 g/L.

### Germination and plant regeneration

Mature cotyledonary embryos from ECL 1-3-5 were transferred to PGR-free solid LP basal medium containing 20 g/L maltose, 2.0 g/L AC and 8 g/L agar to achieve germination. Petri dishes were incubated for the first 3-7 days in darkness. Then, cultures were transferred to a growth chamber, under conditions as described in Yang et al. (2020). When somatic embryos germinated after 1 month, they were aseptically transplanted to a rooting medium which consisted of 0.2 mg/L α-naphthaleneacetic acid (NAA), 1.0 mg/L indole-3-butyric acid (IBA), 10 g/L sucrose, 1.0 g/L myo-inositol, 0.5 g/L AC and 10 g/L carrageenan. Regenerated plantlets were obtained after about 6-8 weeks of culture in the light. Data on germination and percentage rooting were recorded 1 month after transfer to rooting medium.

### Effects of ectomycorrhizal fungi on regenerated plantlets

The seven-month-old regenerated plantlets from three ECLs (20-1-4, 20-1-7 and 20-1-16) were used to carry out inoculation treatments. The fungal isolate used for our experiment was *Pisolithus orientalis*, which was purchased from Agricultural Culture Collection of China (ACCC), Beijing, China. Prior to use, it was cultured on potato dextrose [glucose] agar (PDA) medium. The regenerated plantlets were transplanted into sterilized containers, which contained sterilized pearlite as substrate. Rooted plantlets were inoculated with the mycelium of ectomycorrhizal fungus; regenerated plantlets without ectomycorrhizal fungi were used as control. Plantlets were watered regularly with Gupta and Durzan culture (DCR) solution, with 20 g/L sucrose and 0.5 g/L myo-inositol added. To minimize within-sample variation, regenerated plantlets of uniform size and growth vigor were selected for the trials. For each ECL, 35 regenerated plantlets were mycorrhized and 15 non-mycorrhized, regenerated plantlets were used as control. After six months, the height was recorded and the regenerated plantlets were transferred to plastic pots (10 cm in diameter, 8.5 cm in height) filled with sterile peat, coir, vermiculite, perlite and soil. The regenerated plantlets were acclimatized in a growth cabinet, as described in Sun et al. (2019b). The height and survival rate were recorded 60 days after transfer.

### Infestation of *P. massoniana* regenerated plantlets by *B. xylophilus*

*P. massoniana* regenerated plantlets from three ECLs (20-1-4, 20-1-7 and 20-1-16) grown under sterile environmental conditions were infested with *B. xylophilus*. The aseptic PWN strain used for infestation was the strongly virulent strain AMA3C28 maintained in our laboratory. The nematodes were subcultured on *Botrytis cinerea* colonies on PDA in a 25 °C incubator for further use. The *in vitro* infestation method was as described by Zhu et al. (2011); Zhu et al. (2012). Each treatment plantlet was inoculated with 500 aseptic nematodes, while each control plantlet was inoculated with aseptic water. There were two classes of PWN-infested or control plantlets: inoculated with ectomycorrhizal fungi or not inoculated with the ectomycorrhizal fungus. Each treatment or control group consisted of at least three replicate plantlets. The number of wilted plantlets in each treatment was recorded every 2 days until 45 days after inoculation, at which point, nematodes were recovered by the Baermann funnel method (Staniland, 1954) from the wilted plantlets and the corresponding culture medium. The number of nematodes recovered was counted under a light microscope.

### Statistical analysis

Data were analyzed by GraphPad Prism Software version 8.0.2 and IBM SPSS Statistics 19. All test data were analyzed by analysis of variance (ANOVA) and Duncan’s multiple range test. Each data point represents the mean ± standard error.

## Results

### Initiation, maintenance and proliferation of callus

**Figure 1:**
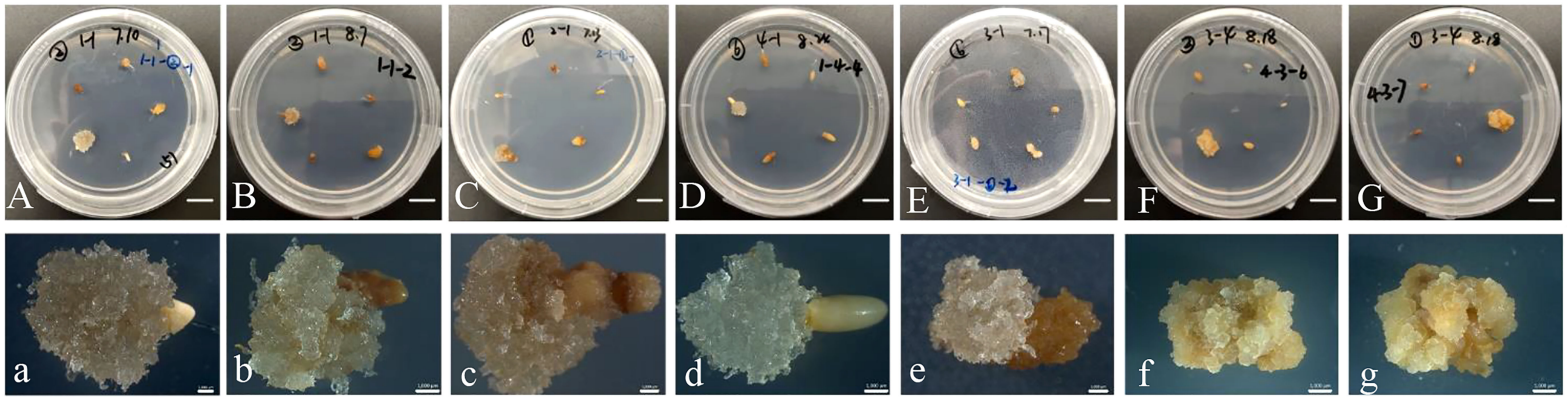
Initiation of callus tissues in *Pinus massoniana* Lamb. **(A, B)** Embryogenic callus produced from seed family GX1 on the number 2 medium (**Table 1**) with PGRs (1.0 mg/L 2,4-D and 1.0 mg/L 6-BA). **(C)** Embryogenic callus produced from seed family GX1 on the number 1 medium with PGRs (1.0 mg/L 2,4-D and 0.5 mg/L 6-BA). **(D, E)** Embryogenic callus produced from seed family GX1 on the number 6 medium with PGRs (2.0 mg/L 2,4-D and 1.0 mg/L 6-BA). **(F)** Embryogenic callus produced from family GX4 on the number 2 medium with PGRs (1.0 mg/L 2,4-D and 1.0 mg/L 6-BA). **(G)** Embryogenic callus produced from family GX4 on the number 1 medium with PGRs (1.0 mg/L 2,4-D and 0.5 mg/L 6-BA). **(a–g)** Characteristics of callus tissues **(A, B)** under the stereomicroscope. **(A–G)** Scale bar = 1.0 cm. **(a–g)** Scale bar = 0.1 cm.

After a two-month of culture period on the initiation medium, calli were successfully obtained. The initiation frequencies varied from 0 to 10% among different families and PGR concentrations. Calli were obtained from explants from seed families GX1 (**Figures 1A–E**) and GX4 (**Figures 1F, G**), with initiation frequencies of 2.3% and 10%, respectively. The remaining families showed no induction response. Among all four families, the number 1 medium (**Table 1**) exhibited the highest initiation response with a mean of 6.7%, followed by the number 2 medium (**Table 1**) with 5.2%. During subculture, different callus types presented varying morphologies: embryogenic tissue was smooth, white and translucent (**Figures 1A–E**), while non-embryogenic callus was friable and compact (**Figures 1F, G**). Embryogenic calli were obtained on proliferation medium. Double staining revealed that the embryogenic callus showed an organization of embryonal head cells which stain bright red (acetocarmine) and suspensor cells stain blue, while, non-embryogenic callus, on the other hand, cells do not show any organization of head and suspensor (**Extra figure 1**).

### Effect of combined treatment with ABA, PEG 8000, AC and maltose on SE maturation and germination

**Figure 2:**
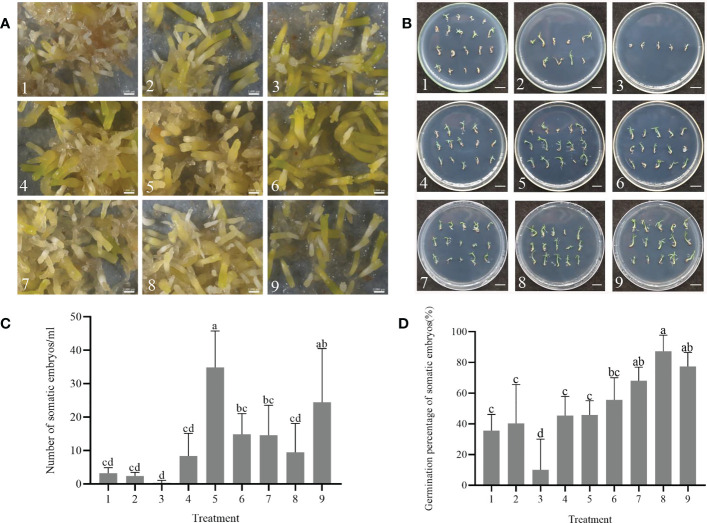
Effect of combined treatments with abscisic acid (ABA), polyethylene glycol 8000 (PEG 8000), activated carbon (AC), and maltose on somatic embryo maturation and germination of *P. massoniana.* **(A)** The callus surface was different degrees of yellow, brown or green color after 75 days. The somatic embryos were visible at different maturation medium compositions under the stereomicroscope. Scale bar = 0.1 cm. **(B)** Somatic embryos germinated for one month. Scale bar = 1.0 cm. **(C)** Number of somatic embryos in different treatments after 75 days of maturation. **(D)** Germination rate of somatic embryos. Data represent mean ± SD of replicates. Different lowercase letters above the bars indicate a significant difference using ANOVA and Duncan’s test (p < 0.05). Numbers represent maturation medium composition supplemented with 1: 1 mg/L ABA, 110 g/L PEG 8000, 0.5 g/L AC and 20 g/L maltose. 2: 1 mg/L ABA, 130 g/L PEG 8000, 1.0 g/L AC and 25 g/L maltose. 3: 1 mg/L ABA, 150 g/L PEG 8000, 1.5 g/L AC and 30 g/L maltose. 4: 2 mg/L ABA, 110 g/L PEG 8000, 0.5 g/L AC and 30 g/L maltose. 5: 2 mg/L ABA, 130 g/L PEG 8000, 1.0 g/L AC and 20 g/L maltose. 6: 2 mg/L ABA, 150 g/L PEG 8000, 1.5 g/L AC and 25 g/L maltose. 7: 3 mg/L ABA, 110 g/L PEG 8000, 0.5 g/L AC and 25 g/L maltose. 8: 3 mg/L ABA, 130 g/L PEG 8000, 1.0 g/L AC and 30 g/L maltose. 9: 3 mg/L ABA, 150 g/L PEG 8000, 1.5 g/L AC and 20 g/L maltose.

After 75 days of culture on solid maturation medium, the calli displayed varying degrees of yellow, brown or green colors with visible somatic embryos (**Figure 2A**). The cotyledonary somatic embryos cultured for 75 days on solid maturation medium were transferred to germination medium and after one month, the embryonic axis had elongated, and the cotyledons gradually opened (**Figure 2B**). The yield of somatic embryos was affected by the concentrations of ABA, PEG 8000, AC and maltose, with production peaking at number 5 treatment (maturation medium supplemented 2 mg/L ABA, 130 g/L PEG, 1.0 g/L AC and 20 g/L maltose), followed by number 9 treatment (maturation medium supplemented 3 mg/L ABA, 150 g/L PEG, 1.5 g/L AC and 20 g/L maltose), with the average number of somatic embryos being 34.9 ± 9.4 per mL and 24.4 ± 13.9 per mL, respectively (**Figure 2C**). Among the nine maturation medium compositions tested, the germination rate of somatic embryos was affected by concentrations of ABA, PEG 8000, AC and maltose, with the highest germination percentage observed at number 8 treatment (maturation medium supplemented with 3 mg/L ABA, 130 g/L PEG, 1.0 g/L AC and 30 g/L maltose), with an average of 87.3 ± 9.1% (**Figure 2D**). **Table 3** shows the correlation between the range of each factor, where R_ABA_ > R _maltose_ > R_AC_ > R_PEG_. This indicates that the concentration of ABA, maltose, AC, and PEG had a primary and secondary order of impact on the number of somatic embryo maturation. The optimal composition of the medium was found to be 2 mg/L ABA, 130 g/L PEG, 1.5 g/L AC and 20 g/L maltose. In **Table 4**, the relationship between the range of each factor was observed to be R_ABA_ > R_AC_ > R_PEG_ > R _maltose_. The optimal solution was found to be 3 mg/L ABA, 150 g/L PEG, 0.5 g/L AC, and 25 g/L maltose.

**Table 3:** Results of the influence of ABA, PEG, AC and maltose on somatic embryo maturation in *P. massoniana*.

Medium	Factors					Quantity of somatic embryos (number per mL)
number		ABA (mg/L)	PEG (g/L)	AC (g/L)	Maltose (g/L)	
>1		1	110	0.5	20	3.2
2		1	130	1.0	25	2.4
3		1	150	1.5	30	0.4
4		2	110	0.5	30	8.4
5		2	130	1.0	20	34.9
6		2	150	1.5	25	14.9
7		3	110	0.5	25	14.6
8		3	130	1.0	30	9.5
9		3	150	1.5	20	24.4
	y_j1_	6	26.2	27.6	62.5	
	y_j2_	58.2	46.8	35.2	31.9	
	y_j3_	48.5	39.7	49.9	18.3	
	${\bar{y}}_{j1}$	2	8.7	9.2	20.8	
	${\bar{y}}_{j2}$	19.4	15.6	11.7	10.6	
	${\bar{y}}_{j3}$	16.2	13.2	16.6	6.1	
	R_j_	17.4	6.9	7.4	14.7	
Primary and secondary factor				ABA>Maltose>AC>PEG		
Optimal composition				ABA2 + PEG2 + AC3 + Maltose1		

ABA, abscisic acid; PEG, polyethylene glycol 8000; AC, activated carbon. y_jk_ (k=1, 2, 3) is the sum of test results with the same level of k in the j_th_ column of the table; ${\bar{y}}_{jk}$ is the mean value of the test results with the same level of k in the j_th_ column of the table; R_j_ is the range of ${\bar{y}}_{jk}$ .R_j_=max( ${\bar{y}}_{jk}$ )- min( ${\bar{y}}_{jk}$ ).

**Table 4:** Results of the influence of ABA, PEG, AC and maltose on somatic embryo germination in *P. massoniana*.

Medium	Factors					Germination rate (%)
number		ABA (mg/L)	PEG (g/L)	AC (g/L)	Maltose (g/L)	
1		1	110	0.5	20	35.5
2		1	130	1.0	25	40.3
3		1	150	1.5	30	10.0
4		2	110	0.5	30	45.4
5		2	130	1.0	20	45.8
6		2	150	1.5	25	55.6
7		3	110	0.5	25	68.1
8		3	130	1.0	30	87.3
9		3	150	1.5	20	68.8
	y_j1_	85.8	149	178.4	150.1	
	y_j2_	146.8	173.4	154.5	164	
	y_j3_	224.2	134.4	123.9	142.7	
	${\bar{y}}_{j1}$	28.6	49.7	59.5	50.0	
	${\bar{y}}_{j2}$	48.9	57.8	51.5	54.7	
	${\bar{y}}_{j3}$	74.7	44.8	41.3	47.6	
	R_j_	46.1	13	18.2	7.1	
Primary and secondary factors				ABA>AC>PEG>Maltose		
Optimal composition				ABA3 + PEG2 + AC1 + Maltose2		

ABA, abscisic acid; PEG, polyethylene glycol 8000; AC, activated carbon. y_jk_ (k=1, 2, 3) is the sum of test results with the same level of k in the j_th_ column of the table; ${\bar{y}}_{jk}$ is the mean value of the test results with the same level of k in the j_th_ column of the table; R_j_ is the range of ${\bar{y}}_{jk}$ . R_j_=max( ${\bar{y}}_{jk}$ )- min( ${\bar{y}}_{jk}$ ).

### Effect of PEG 8000 concentration on somatic embryo maturation and germination

**Figure 3:**
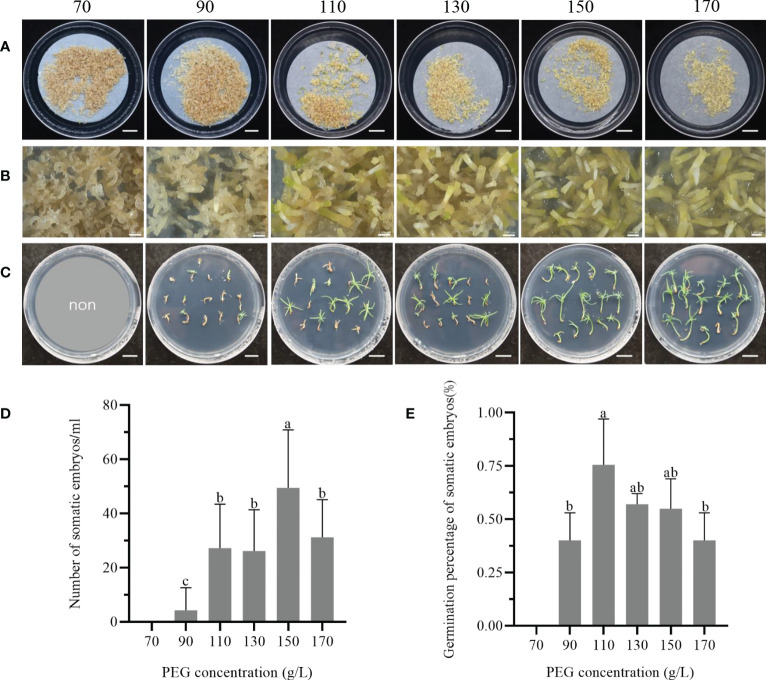
Effect of PEG concentration in the maturation medium on the maturation and germination of somatic embryos of *P. massoniana*. **(A)** The concentrations of PEG from left to right are 70, 90, 110, 130, 150 or 170 g/L in solid culture, and somatic embryos were obtained after 75 days. Scale bar = 1.0 cm. **(B)** was a further close-up of **(A)** under the stereomicroscope. Scale bar = 0.1 cm. **(C)** Germination of masson pine somatic embryos on germination medium after 1 month. Scale bar = 1.0 cm. **(D)** Number of somatic embryos obtained from solid maturation medium after 75 days. **(E)** Germination rate of somatic embryos. Data represent the mean ± SE of the sample. Different lowercase letters above the bars indicate significant differences according to ANOVA and Duncan’s test (p < 0.05).

The addition of PEG to the maturation medium was found to enhance the ability of somatic embryos during culture. **Figure 3A** illustrated the callus surface colour from dark brown to light yellow-white in response to increasing PEG concentration. The embryogenic cell proliferation was observed to be active on medium containing 70 and 90 g/L PEG (**Figure 3B**), whereas many poorly developed embryos were seen on media containing 110 and 130 g/L PEG. However, when the PEG concentration in the medium was increased to 150 - 170 g/L, a significant number of mature cotyledonary embryos were observed. Following 75 days of culturing somatic embryos on maturation media with varying PEG concentrations, they were transferred to the germination medium and germinated under standard conditions (**Figure 3C**).

The number of mature cotyledonary embryos initially increased and then decreased in response to the concentration of PEG in the maturation medium. The highest cotyledonary embryo production was observed at 150 g/L PEG, followed by a significant decrease in the number of mature cotyledonary embryos when the PEG concentration was further increased to 170 g/L (**Figure 3D**). Furthermore, the germination percentage of somatic embryos varied among media with different PEG concentrations. Somatic embryos cultured in 110 g/L PEG exhibited a germination rate of 75.3 ± 21.6% (**Figure 3E**), but showed weak growth. Conversely, when the concentration of PEG in the maturation medium was increased to 150 or 170 g/L, the germination and development of the somatic embryos were improved, resulting in longer hypocotyls and more open cotyledons. To achieve strong plantlets, the optimum concentration range of PEG in the maturation medium was found to be 150 - 170 g/L.

### Plantlet regeneration

**Figure 4:**
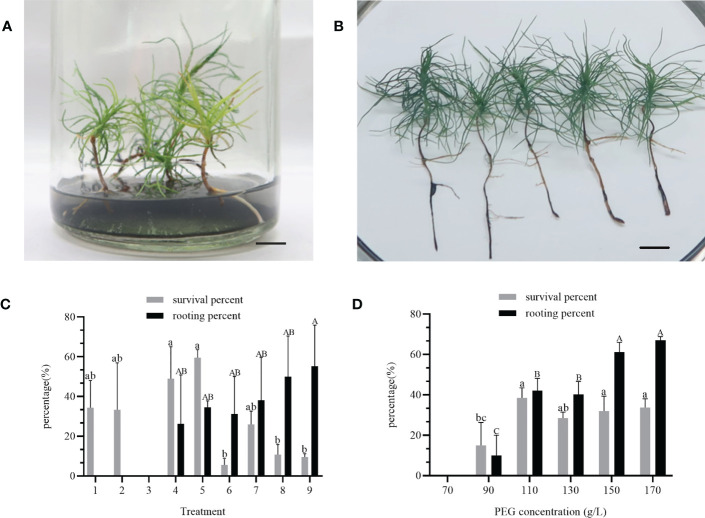
Plantlet regeneration from cell line 1-3-5 of *P. massoniana*. **(A)** Somatic embryo regenerated plantlets on rooting medium for 2 months, developed white root tips. Scale bar = 1.0 cm. **(B)** Plantlets regenerated under light conditions, showing well-developed shoot and roots (4 months). Scale bar = 1.0 cm. **(C)** Percentage survival and rooting of plantlets after different maturation treatments on somatic embryos (combined treatments of different concentrations of abscisic acid (ABA), poly-ethylene glycol 8000 (PEG), activated carbon (AC) and maltose) of *P. massoniana*. **(D)** Survival and rooting rates of plantlets after maturation treatment with different concentrations of PEG (70, 90, 110, 130, 150 or 170 g/L) on somatic embryos of *P. massoniana*. Data represent the mean ± SD of replicates. Different letters above the bars of the same color indicate significant differences ac-cording to ANOVA and Duncan’s test (p < 0.05).

After two months of culture on rooting medium, complete regeneration of plantlets with white root tips was observed (**Figure 4A**). After four months of culture, the shoot and roots were well-developed, and the needles exhibited vigorous growth (**Figure 4B**). The survival and rooting percentages of regenerated plantlets were significantly affected by the maturation treatments. The highest survival percentage of 59.6% was observed in treatment number 5 treatment, which involved maturation medium supplemented with 2 mg/L ABA, 130 g/L PEG, 1.0 g/L AC and 20 g/L maltose (**Figure 4C**). However, the rooting percentage in this treatment was only 35.0%. Treatment number 9 showed the highest rooting percentage of 55.2%, but no significant differences were observed among the other treatments. The concentration of PEG in maturation medium significantly affected both the survival and the rooting in plantlets. After four months, the survival percentages of the plantlets in response to PEG concentration in the maturation treatment varied significantly different, with the highest survival rate at 38.5% and the lowest 15.0% (**Figure 4D**). Furthermore, increasing the PEG concentration in the maturation medium led to a higher rooting percentage, with the highest rooting percentage observed at 170 g/L PEG, reaching up to 66.9%.

The result of the range analysis revealed that the range of PEG had the most significant effect on plantlet survival in the maturation treatments, followed by ABA, maltose, and AC (**Table 5**). The optimal solution for plantlet survival was found to be 2 mg/L ABA, 130 g/L PEG, 1.0 g/L AC, and 20 g/L maltose. Similarly, for plantlet rooting percentage, ABA was found to be the most important factor, followed by maltose, AC, and PEG (**Table 6**). The optimal solution for plantlet rooting percentage was found to be 3 mg/L ABA, 170 g/L PEG, 1.0 g/L AC, and 30 g/L maltose. These findings indicate that the maturation treatment altered the plantlet rooting response to the composition of the rooting medium, which necessitated the addition of varying concentrations of ABA and PEG to the medium.

**Table 5:** The influence of ABA, PEG, AC and maltose on plantlet survival of *P. massoniana*.

Medium		Factors				Survival rate (%)
number		ABA (mg/L)	PEG (g/L)	AC (g/L)	Maltose (g/L)	
1		1	110	0.5	20	34.4
2		1	130	1.0	25	33.3
3		1	150	1.5	30	0
4		2	110	0.5	30	49.0
5		2	130	1.0	20	59.6
6		2	150	1.5	25	5.6
7		3	110	0.5	25	26.0
8		3	130	1.0	30	10.7
9		3	150	1.5	20	9.6
	y_j1_	67.7	109.4	50.7	103.6	
	y_j2_	114.2	103.6	91.9	64.9	
	y_j3_	46.3	15.2	85.6	59.7	
	${\bar{y}}_{j1}$	22.6	36.5	16.9	34.5	
	${\bar{y}}_{j2}$	38.1	34.5	30.6	21.6	
	${\bar{y}}_{j3}$	15.4	5.1	28.5	19.9	
	R_j_	22.7	31.4	13.7	14.6	
Primary and secondary factors				PEG>ABA>Maltose>AC		
Optimal concentration				ABA2+PEG1+AC2+Maltose1		

ABA, abscisic acid; PEG, polyethylene glycol 8000; AC, activated carbon. y_jk_ (k=1, 2, 3) is the sum of test results with the same level of k in the j_th_ column of the table; ${\bar{y}}_{jk}$ is the mean value of the test results with the same level of k in the j_th_ column of the table; R_j_ is the range of ${\bar{y}}_{jk}$ . R_j_=max( ${\bar{y}}_{jk}$ )- min( ${\bar{y}}_{jk}$ ).

**Table 6:** Influence of ABA, PEG, AC and maltose concentration on plantlet rooting in *P. massoniana*.

Medium		Factors				Rooting rate (%)
number		ABA (mg/L)	PEG (g/L)	AC (g/L)	Maltose (g/L)	
1		1	110	0.5	20	0
2		1	130	1.0	25	0
3		1	150	1.5	30	0
4		2	110	0.5	30	35.0
5		2	130	1.0	20	34.6
6		2	150	1.5	25	31.3
7		3	110	0.5	25	38.1
8		3	130	1.0	30	50.0
9		3	150	1.5	20	55.2
	y_j1_	0	73.1	81.3	89.8	
	y_j2_	100.9	84.6	90.2	69.4	
	y_j3_	143.3	86.5	72.7	85	
	${\bar{y}}_{j1}$	0	24.4	27.1	29.9	
	${\bar{y}}_{j2}$	33.6	28.2	30.1	23.1	
	${\bar{y}}_{j3}$	47.8	28.8	24.2	51.7	
	R_j_	47.8	4.4	5.9	28.6	
	Primary and secondary factors			ABA>Maltose>AC>PEG		
	Optimal composition			ABA3+PEG3+AC2+Maltose3		

ABA, abscisic acid; PEG, polyethylene glycol 8000; AC, activated carbon. y_jk_ (k=1, 2, 3) is the sum of test results with the same level of k in the j_th_ column of the table; ${\bar{y}}_{jk}$ is the mean value of the test results with the same level of k in the j_th_ column of the table; R_j_ is the range of ${\bar{y}}_{jk}$ . R_j_=max( ${\bar{y}}_{jk}$ )- min( ${\bar{y}}_{jk}$ ).

### Effects of ectomycorrhizal fungi on regenerated plantlets

**Figure 5:**
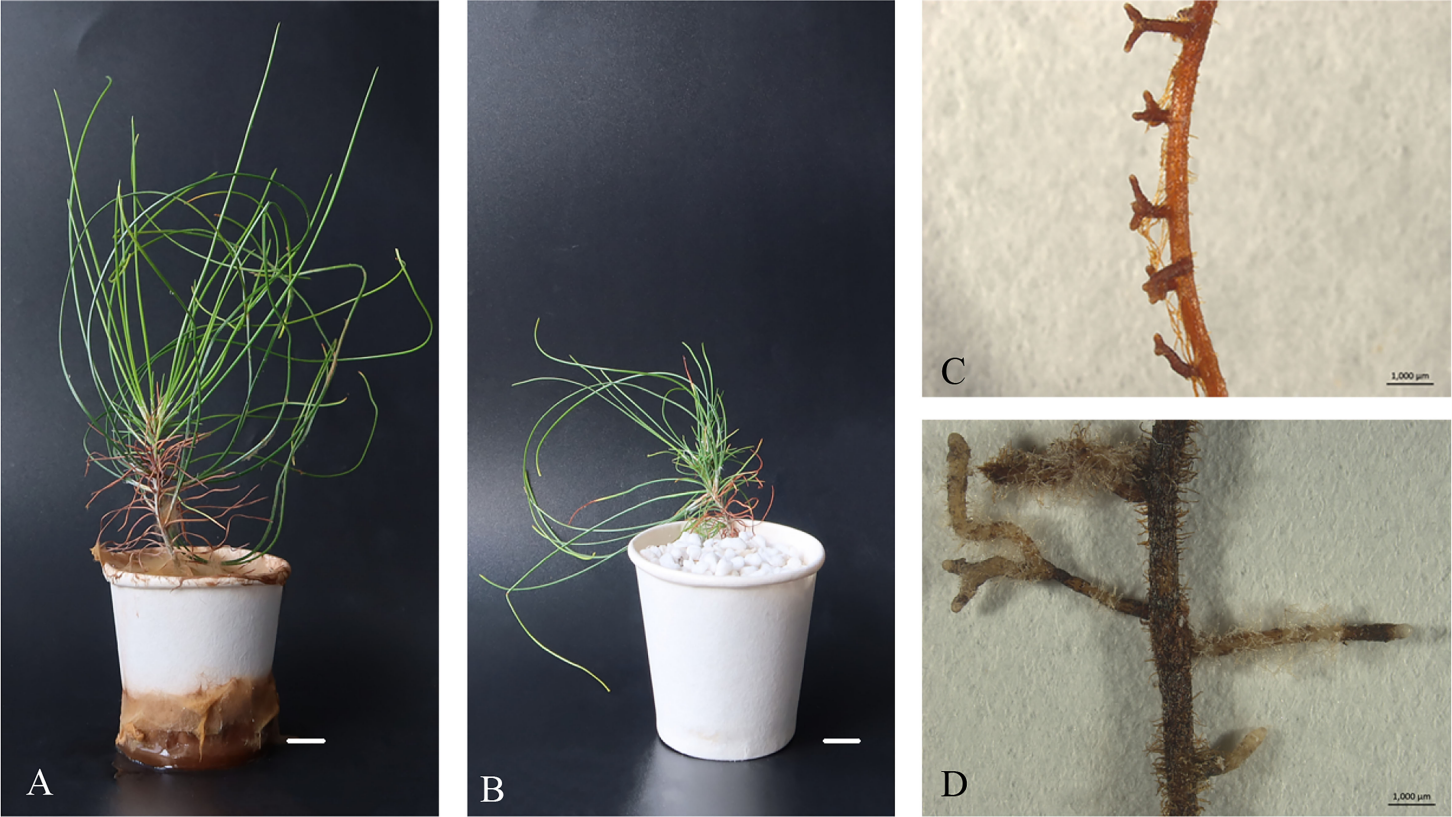
Morphology of *P. massoniana* ectomycorrhiza in regenerated plantlets. **(A)** Plantlets after inoculation with the ectomycorrhizal fungus *Pisolithus orientalis*. Scale bar = 1.0 cm. **(B)** Uninoculated plantlets. Scale bar = 1.0 cm. **(C)** Details of a root with dichotomously branched, ectomycorrhiza-like structures viewed under a Leica MZ16 stereomicroscope. Scale bar = 0.1 cm. **(D)** Non-inoculated root. Scale bar = 0.1 cm.

**Figure 6:**
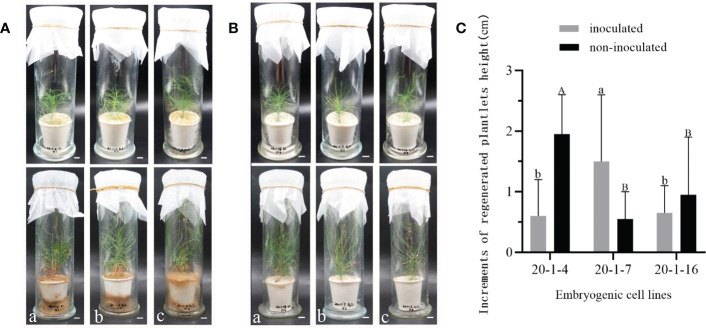
Growth of regenerated plantlets of *P. massoniana* with or without inoculation with the ectomycorrhizal fungus *Pisolithus orientalis*. **(A)** Symptoms of *P. massoniana* plantlets one day (upper) or six months (lower) after inoculation with ectomycorrhizal fungi. Different lowercase letters **(a–c)** represent cell lines 20-1-4, 20-1-7 and 20-1-16, respectively. Scale bar = 1.0 cm. **(B)** Symptoms of uninoculated (control) plantlets. Different lowercase letters **(a–c)** represent cell lines 20-1-4, 20-1-7 and 20-1-16, respectively. Scale bar = 1.0 cm. **(C)** Increments of regenerated plantlet height were recorded after six months. Data represent the mean ± SD of replicates. Different letters above the bars of the same color indicate significant differences according to ANOVA and Duncan’s test (p < 0.05).

The perlite substrate of inoculated *Pis. orientalis* plantlets was rapidly colonized by the fungus (**Figure 5A**), while control, non-inoculated plantlets grew normally (**Figure 5B**). Six months after inoculation, numerous dichotomously branched ectomycorrhiza-like structures were observed (**Figure 5C**). Inoculated plantlet roots were covered by a yellow hyphal coat, and no root hairs were observed on their surface (**Figure 5C**). In contrast, uninoculated control plantlet roots exhibited root hairs and appeared dark in color, but no dichotomously branched structures were present (**Figure 5D**). The height of regenerated plantlets under inoculated (**Figure 6A**) and non-inoculated (**Figure 6B**) conditions varied significantly among different cell lines. Ectomycorrhizal inoculation increased the height of cell line 20-1-7 plantlets by 1.5 cm, while non-inoculated plantlets of cell lines 20-1-4 and 20-1-16 were taller than their corresponding mycorrhized plantlets, with increases being 2.0 and 1.0 cm, respectively (**Figure 6C**).

**Figure 7:**
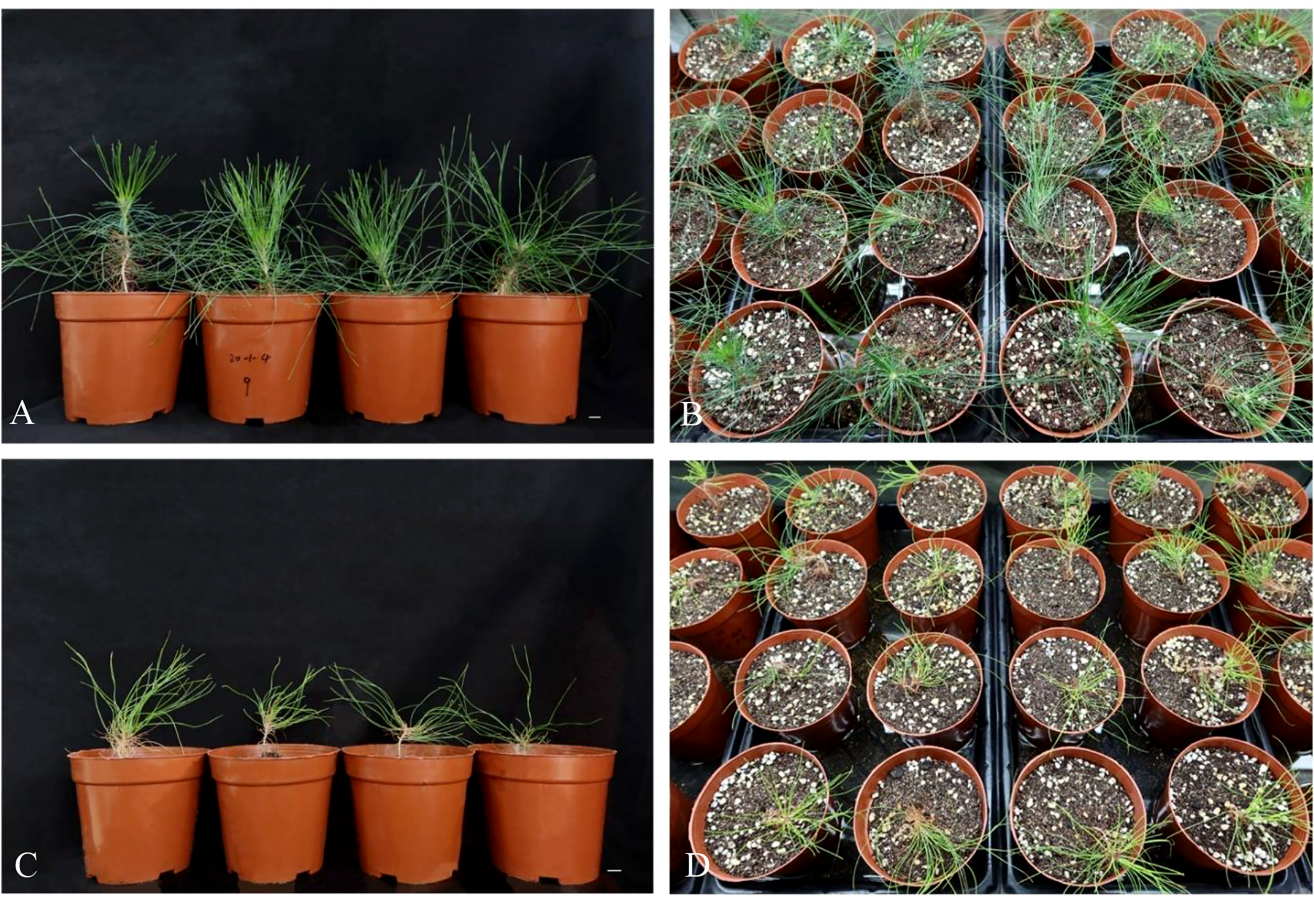
Regenerated plantlets of *P. massoniana* growing in the greenhouse after four months of acclimatization culture. Two types of plantlets are shown, mycorrhizal-inoculated or non-inoculated. **(A, B)** Plantlets after inoculation with the ectomycorrhizal fungus *Pisolithus orientalis*. **(C, D)** Uninoculated (control) plantlets. Scale bar = 1.0 cm.

The regenerated plantlets were transferred from tissue-cultured containers on sterile medium to plastic filled with non-sterile soil for acclimatization (**Figure 7**). After four months, mycorrhized plantlets had green needles (**Figures 7A, B**), whereas control, uninoculated plantlets appeared yellow-green (**Figures 7C, D**). The survival rate of regenerated plantlets in response to mycorrhizal symbiosis was 85%, which was significantly higher than the survival rate of regenerated plantlets without ectomycorrhizal inoculation, which was only 37%.

### The PWN resistance of regenerated *P. massoniana* plantlets

**Figure 8:**
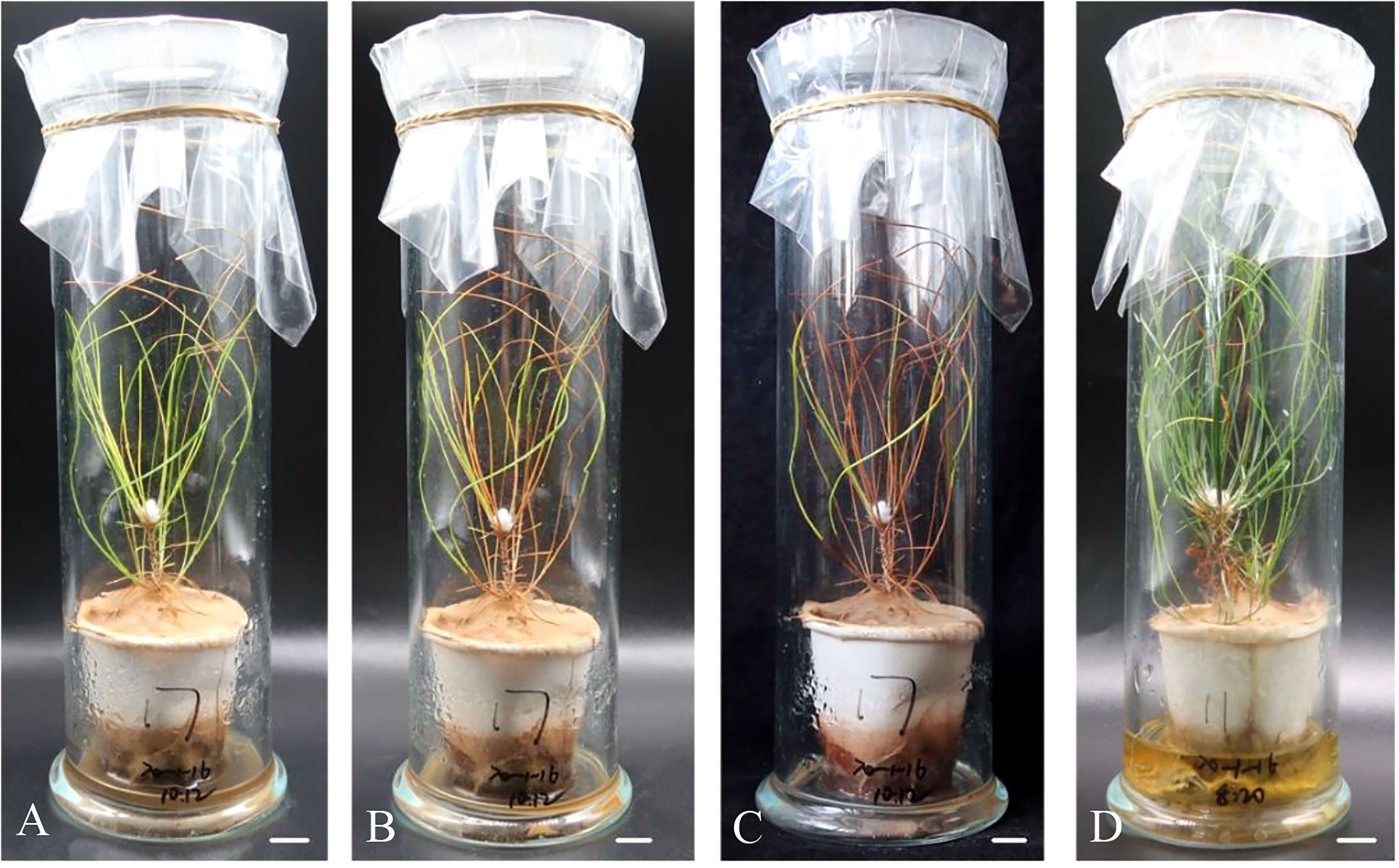
Symptoms of regenerated plantlets *P. massoniana* after inoculation with the aseptic pine wood nematodes. Symptoms of *P. massoniana* at 15 d **(A)**, 25 d **(B)**, and 45 d **(C)** after inoculation, respectively. **(D)** Control (inoculated with aseptic water) at 45 (d) Scale bar = 1.0 cm.

The regenerated plantlets of all cell lines that were infested with aseptic PWNs showed symptoms similar to those observed in naturally infected pine trees. In the infested plantlets, the needles gradually changed from green to yellow (**Figure 8A**), gradually turning reddish brown without falling off the plant (**Figure 8B**), and eventually the whole plantlets withered and died (**Figure 8C**). Regenerated plantlets that were inoculated with sterile water remained healthy (**Figure 8D**). The time from infestation to the appearance of the first symptoms varied among different cell lines. The first symptoms appeared 11 days post-infestation in non-mycorrhizal plantlets of ECL 20-1-4 (**Table 7**), and 13 days and 24 days in the corresponding non-mycorrhizal plantlets of ECLs 20-1-7 and 20-1-16, respectively. The time from infestation to the appearance of the first symptoms of mycorrhizal plantlets of ECLs 20-1-4, 20-1-7 and 20-1-16 were 12 days, 13 days and 16 days, respectively (**Table 7**).

**Table 7:** Wilting rates of mycorrhizal and non-mycorrhizal plantlets of *P. massoniana* cell lines after infestation with sterile pine wood nematodes and number of nematodes recovered from the wilted plantlets (45 d after infestation).

Host plantlets	Number	Time of first symptoms (d)	Wilting ratio (%)	Recovery of nematodes
20-1-4-A	12	11	50	4445 ± 587
20-1-4-B	12	12	25	8575 ± 1716
20-1-7-A	12	13	8.3	272
20-1-7-B	12	13	8.3	625
20-1-16-A	15	24	40	4552 ± 3115
20-1-16-B	15	16	6.7	608
CK-A	9	–	–	N
CK-B	9	–	–	N

Data points represent the mean ± SD. “A” means non-mycorrhizal plantlets. “B” means mycorrhizal plantlets. “N’’ means “not done”. “-” means “none”. “CK” means control (aseptic water).

The wilting rate in response to infestation with aseptic PWNs varied greatly among the different cell lines 45 days after inoculation. Non-mycorrhized plantlets of ECLs 20-1-4, 20-1-7 and 20-1-16 had wilting percentages of 50%, 8.3%, and 40%, respectively, after infestation with aseptic PWNs, while mycorrhized plantlets of the same cell lines had ratios of 25%, 8.3%, and 6.7%, respectively (**Table 7**). The difference in resistance between the cell lines was apparent, with ECL 20-1-7 showing the greatest resistance and 20-1-4 showing the least resistance to PWNs. Nematodes were recovered from both wilting non-mycorrhizal and mycorrhizal plantlets that were infested with aseptic PWNs. There were significant differences in the number of nematodes recovered between the two types of infested plantlets of ECLs 20-1-4 and 20-1-7. The number of nematodes recovered from wilting re-generated plantlets was higher in the mycorrhizal plantlets than in the non-mycorrhizal plantlets; however, in plantlets of ECL 20-1-16, the opposite effects were observed, with more nematodes in the non-mycorrhized plantlets (**Table 7**). The average numbers of nematodes recovered from wilting non-mycorrhizal plantlets in ECLs 20-1-4, 20-1-7 and 20-1-16 were 4445, 272 and 4552, respectively, compared with 8575, 625 and 608, respectively, in mycorrhizal plantlets (**Table 7**).

## Discussion

In continuation of Xia et al. (2021), we successfully obtained embryogenic callus of *P. massoniana*. Our study revealed that different seed families exhibited significantly different capacities for embryonal mass initiation, ranging from 0% to 10%. Consistent with Xia et al. (2021), we found that families GX1 and GX4 were more responsive than families GX2 and GX3, highlighting the importance of selecting appropriate mother trees to increase the likelihood of obtaining desired genotypes (Carneros et al., 2009). Future studies could explore a wider range of mother tree types at the embryo initiation stage. Similar to previous reports by Kim and Moon (2007); Hosoi and Maruyama (2012), and Sun et al. (2019b), we found that not all callus could survive and proliferate on proliferation medium. In our experiments, few embryogenic calli survived and proliferated on proliferation medium. Multiple factors influence somatic embryos maturation, including genotype, PGRs, induction treatments, basal medium and medium supplements (Pullman and Skryabina, 2007; Vales et al., 2007; Pullman and Johnson, 2009; Pullman and Bucalo, 2014; Tang et al., 2020). In our study, we focused on four factors that potentially influence *P. massoniana* SE maturation, namely ABA, PEG 8000, AC, and maltose. We conducted multifactorial experiments on these factors to identify their primary and secondary relationships in the processes of SE maturation, germination, survival, and rooting.

Exogenous ABA is known to play a crucial role in regulating the formation and development of somatic embryos, as reported by several studies (Wang et al., 2002; Carneros et al., 2017; Zhang et al., 2019). PEG has been found to induce water stress that mimics drought conditions, thus reducing the water content of somatic embryos and promoting their maturation at an appropriate concentration (Shoji et al., 2006). AC, on the other hand, is believed to adsorb waste and toxic substances from the culture medium (Pullman et al., 2005). Apart from serving as a carbon source, maltose is also used as a penetrant in cell cultures (Xia et al., 2021). Our study used a multifactorial test, which revealed that ABA was the main factor affecting the maturation of somatic embryos in *P. massoniana*, while maltose was the secondary factor, similar to the findings of Liao and Amerson (1995). Addition of ABA has been reported to promote the maturation in somatic embryos in other pine species (Wang et al., 2002; Yang et al., 2020), but the required ABA concentration varies considerably between tree species. Sun et al. (2019a) found that 20 mg/L ABA maturation treatment increased the number of somatic embryos in *P. thunbergii*, while Yang et al. (2020) found that 5 mg/L ABA in suspension culture significantly increased somatic embryo production in *P. elliottii*. Furthermore, the response of SE to exogenous ABA is known to be genotype-dependent in some conifers (Kong and von Aderkas, 2007; Carneros et al., 2017). The promotion of SE formation by maltose has also been reported in other studies (Sun et al., 2019a; Xia et al., 2021).

The germination of somatic embryos can be influenced by various factors, including the maturation treatment (Salaj et al., 2019). The results of the current study indicate that the primary factor affecting the subsequent germination of somatic embryos in *P. massoniana* was ABA, followed by AC. The maximum germination rate was observed to be 87.3 ± 9.1%. The combination of ABA and AC was found to further promote the formation of cotyledon embryos, which is consistent with previous studies (Huang et al., 1995). Yao and Wang (2020) have reported that AC supplementation in the medium is important for the germination of mature somatic embryos, but excessive amounts of AC can have an inhibitory effect on germination. In our study, we used different amounts of AC in the maturation medium, which could have contributed to the observed variation in the germination rate of subsequent somatic embryos.

Shoji et al. (2006) investigated the effect of osmotic pressure (as influenced by ABA, PEG and maltose) on somatic embryo maturation in *P. densiflora* when the somatic embryos were transferred to a hormone-free medium, the cotyledons developed normally, and the hypocotyls elongated, but rooting was suppressed in all embryos tested. Therefore, we investigated the effects of maturation treatments on embryo survival and rooting. The primary factor affecting the subsequent survival of somatic embryos was PEG, followed by ABA, with a survival rate up to 59.6 ± 6.8%. ABA was the primary factor affecting the subsequent rooting of somatic embryos, with maltose being the secondary factor. The rooting rate reached up to 55.2 ± 29.3%. The low rooting rate was similar to that identified by Salaj et al. (2019), which showed that maturation treatment affected somatic embryo germination. Based on the above analysis, we found that the concentration of ABA added in the maturation treatment had a continuing impact on the subsequent growth of *P. massoniana* embryos, which may be associated with changes in the expression of some specific genes (Song et al., 2018). In summary, the best effects on subsequent SE of *P. massoniana* were obtained with 2 - 3 mg/L ABA, 130 - 150 g/L PEG 8000, 1 - 1.5 g/L AC and 20 g/L maltose added to the maturation medium. The optimum composition of this medium differed from that of Xia et al. (2021), reflecting differences in SE capacity between genotypes (Diaz-Sala, 2019). Somatic embryo development was dependent on the cell line (Salajova et al., 1999).

The somatic embryo germination of *P. massoniana* in the multifactorial test was predominantly low and survival was also low, Therefore, we conducted a single-factor test on PEG, which is the main factor affecting the survival of somatic embryos. As PEG concentration increased, the number of mature cotyledonary embryos initially increased and then decreased. These results were consistent with those reported Zhou et al. (2017); Li et al. (2022a). Higher stable osmolarity and the appropriate molecular weight of PEG are key factors for the production of high-quality somatic embryos (Shoji et al., 2006). In our study, the germination rate of somatic embryos tended to increase and then decrease in response to the increase in PEG concentration in the maturation medium. The highest germination rate of somatic embryos (75.3 ± 21.6%) was achieved when 110 g/L PEG was added. Interestingly, somatic embryo plantlets treated with low concentrations of PEG in the maturation medium exhibited better survival rates, whereas those treated with high concentrations of PEG rooted better. This phenomenon had not been reported previously, as earlier studies primarily focused on the impact of PEG on maturity (Jiang et al., 2021). It should be noted that the results from one cell line tested may not be generalizable, and more cell lines need to be studied in subsequent experimentation. However, adding different concentrations of PEG during somatic embryo maturation and observation of the subsequent growth may be a viable method for directly screening the best culture protocol of SE for experimental purposes.

Under aseptic conditions, the survival rate of *in vitro* grown plantlets was low due to the absence of ectomycorrhizal fungi (Wang et al., 2015). Inoculating mycorrhizal fungi can promote the growth of *in vitro* grown seedlings, and improve the adaptability of the *in vitro* regenerated plantlets to the transplant environment (Niemi and HäGgman, 2002; Zhu et al., 2010). Wang et al. (2015) reported that the mycorrhizal fungi *Rhizopogen luteous*, *Pisolithus tinctorius* and *Boletus edulis* were effective in improving the survival rate of transplanted cultured plantlets of *P. densiflora*. Niemi et al. (2007) showed that using specific ectomycorrhizal fungi can promote plant development, but not root formation. In our study, we also observed that inoculation with an ectomycorrhizal fungus promoted plant development. Ectomycorrhizal inoculation increased the height of regenerated plantlets of cell line 20-1-7, but this effect was not evident in the other two cell lines. The effects of inoculation on the growth of cell lines varied from cell line to cell line, as reported by Vaario et al. (2015). Yamanaka et al. (2012) demonstrated that growth did not change immediately after mycorrhizal inoculation, but positive effects on root-to-crown ratio and root density were found at five months after inoculation. Only one of the three cell lines responded positively to growth after mycorrhizal inoculation in the current study. The reason for this may be the short growth period before transplanting. All ectomycorrhizal plantlets showed lush growth of needles and leaves after transplanting, and mycorrhization also improved the survival rate of transplants. This finding was consistent with the research results of Oliveira et al. (2003); Wang et al. (2015).

PWD is a very complex disease, and in-vitro inoculation using aseptic nematodes and host plantlets grown aseptically has been proven suitable for accurate study of this plant-pathogen interaction (Zhu et al., 2012). Zhu et al. (2012) demonstrated that inoculation with aseptic PWNs caused wilting of young pine microcuttings and seedlings, and Faria et al. (2015); Lin et al. (2017) and Zhu et al. (2020) verified that aseptic PWNs are pathogenic. In this study, inoculation with aseptic PWNs caused wilting of both non-mycorrhizal and mycorrhizal plantlets, confirming that aseptic PWNs demonstrate pathogenicity. However, the results of this study differed from those of other studies (Han et al., 2003; Wang et al., 2018). The resistance of non-mycorrhizal and mycorrhizal plantlets of *P. massoniana* to PWN was studied in this experiment. One cell line (20-1-7) showed relatively high resistance levels to PWN. Both of the wilting rate and the number of nematodes recovered from ECL 20-1-7 were significantly lower than those from ECLs 20-1-4 and 20-1-16.

Ectomycorrhizal fungi are known to form symbioses relationships with plant roots to promote nutrient uptake, but it remains unclear whether they also induce disease resistance in plants (Wang et al., 2022). Some studies, including Wu et al. (2007) and Deng et al. (2017), have suggested that ectomycorrhizas may improve the resistance of plants to disease, while Wang et al. (2022) suggested that ectomycorrhizal colonization triggers a systemic defense response, enhancing the resistance of pine seedlings to *Sphaeropsis* shoot blight. In the current study, we found that the wilting ratios of non-mycorrhizal regenerated plantlets in all cell lines were significantly higher than those of mycorrhizal regenerated plantlets, indicating that ectomycorrhizas can improve resistance against PWD and delay the death of regenerated pine plantlets inoculated with *B. xylophilus*. This result suggests that ectomycorrhizal fungi may induce disease resistance. However, to obtain more detailed information, stronger supporting evidence using modern molecular biology methods is needed. Currently, the reason for the inhibition of nematode propagation in cell line 20-1-7 and in mycorrhized plantlets in this study is unclear, and further research is required.

## Conclusion

In this study, we investigated the primary and secondary factors affecting the maturation of *P. massoniana* somatic embryos. Additionally, we examined the effects of supplements added to the maturation medium on the subsequent germination, survival and rooting of *P. massoniana* somatic embryos. The regenerated plantlets were then inoculated with ectomycorrhizal fungi and acclimated for transplanting. Our results showed that inoculation with aseptic PWNs caused wilting of non-mycorrhizal and mycorrhizal plantlets of *P. massoniana*. However, the proportion of wilting plants from mycorrhizal regenerated plantlets in all cell lines was lower than that from non-mycorrhizal regenerated plantlets. This plantlet regeneration system and mycorrhization method developed in this study could be applied to produce nematode-resistant plantlets on a large-scale and to further study the interaction between nematode, pines, and mycorrhizal fungi.

## Data availability statement

The original contributions presented in the study are included in the article/**Supplementary Material**. Further inquiries can be directed to the corresponding author.

## Author contributions

Y-MC, L-HZ and J-RY conceived and designed the study. Y-MC, QF, X-RX, and XK performed the experiments and carried out the statistical analysis. Y-MC wrote the manuscript. All authors contributed to manuscript revision and have read and approved the submitted version. All authors contributed to the article and approved the submitted version.
