# Supplementary material for: Pinus massoniana somatic embryo maturation, mycorrhization of regenerated plantlets and its resistance to Bursaphelenchus xylophilus
Source: Front Plant Sci. 2023 May 9;14:1130471. doi: 10.3389/fpls.2023.1130471 (PMC10203517; doi:10.3389/fpls.2023.1130471)
Supplement: Supplementary file 1 [file DataSheet_1.docx]

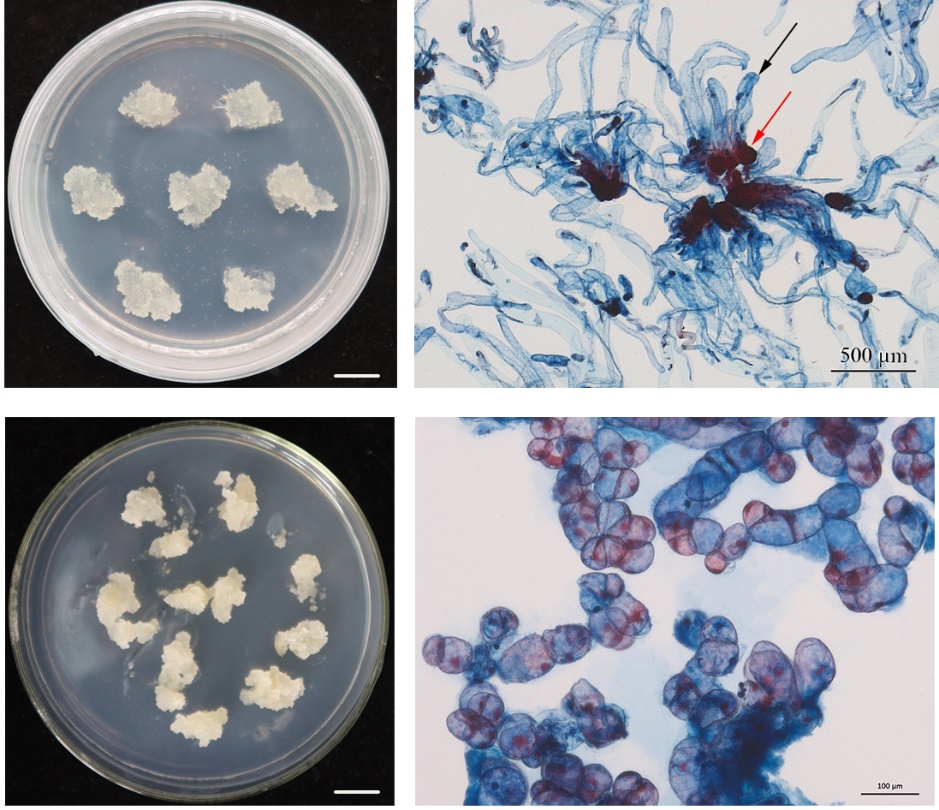


**EXTRA FIGURE 1 |** Maintenance and proliferation of callus with different embryogenic responses, embryo-genic, and non-embryogenic responses in *P. massoniana*. **(A)** Embryogenic callus with a white, translucent appearance. Scale bar = 1.0 cm. **(B)** Embryogenic callus double stained with acetocarmine and Evans blue, red arrows indicate cluster of embryonic head cells, black arrows indicate suspensor cells. Scale bar = 0.05 cm. **(C)** Non-embryogenic callus with a yellow, compact and friable appearance. Scale bar = 1.0 cm. **(D)** Non-embryogenic callus double stained with acetocarmine and Evans blue. Scale bar = 0.01 cm.
